# Phytochemical profiles of lemon verbena (*Lippia citriodora* H.B.K.) and its potential application to cookie enrichment

**DOI:** 10.1002/fsn3.2268

**Published:** 2021-03-29

**Authors:** Abdollah Hematian Sourki, Askar Ghani, Farzaneh Kiani, Azar Alipour

**Affiliations:** ^1^ Department of Food Science and Technology Faculty of Agriculture Jahrom University Jahrom Iran; ^2^ Department of Horticultural Science Faculty of Agriculture Jahrom University Jahrom Iran; ^3^ Department of Food Science and Technology Faculty of Agriculture Shiraz University Shiraz Iran; ^4^ Department of Food Safety and Hygiene School of Public Health Shahid Sadoughi University of Medical Science Yazd Iran

**Keywords:** antioxidant potential, gas chromatography, oxidative stability, polyphenolic compounds, sensory evaluation

## Abstract

In this research, phytochemical properties of lemon verbena and oxidative stability of the fat component in cookies (contain lemon verbena powder and EO) were investigated. The essential oil (EO) profile and polyphenol compounds were identified by GC/MS and HPLC, respectively. Different concentrations of lemon verbena powder and EO were added to the cookies in comparison with TBHQ. The oxidative stability of fat component in cookies (peroxide value, p‐Anisidine, TOTOX value), along with the physicochemical (pH, acidity, weight loss, and moisture content) and sensory properties of the cookies were evaluated over a period of 6 months during storage at room temperature. The main constituents of EO are geranial (27.21%), neral (20.01%), spathulenol (7.28%), and limonene, while trans‐Ferulic acid (6.71 mg/g), Hesperidin (1.87 mg/g), and ρ‐Coumaric acid (0.04 mg/g) were measured as main phenolic compounds. The peroxide value increased in all samples for the first 2 months of storage and then decreased as hydroperoxide was converted to secondary oxidation products. The p‐Anisidine value increased in all samples during storage. This parameter was lower in cookies containing lemon verbena EO and TBHQ treatments. Sensory evaluations of cookies showed that lemon verbena EO had positive effects on the aroma and taste of cookies during storage, whereas lemon verbena powder had adverse effects on mouthfeel and consumer acceptance. The results showed that lemon verbena can increase the eating quality, prolong the shelf life, and maintain the integrity of bakery products with high‐fat content.

## Graphical abstract

Lemon verbena extracts were rich in polyphenols, flavones and flavonols. Thermal stability of the cosokies fat enriched with lemon verbena was improved. By‐products functional ingredients like medicinal plant components may improve technological and sensorial properties.

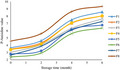

## 1 INTRODUCTION

Cookies are a group of widely used bakery products because of their cheapness, availability, and quick release of energy. Due to their low moisture content, cookies have a relatively long shelf life compared to other bakery products. However, an important and significant issue in relation to the shelf life of cookies is oxidative rancidity of their fat during storage, which has adverse effects on nutritional and sensory qualities. Storing cookies at temperatures above 40°C for 2 months can lead to the rancidity of their fat content (Parcerisa et al., 1999). Through the progress of rancidity and by‐products of oxidative damage, cookies can develop unfavorable flavors, odors, and colors (Park & Pezzuto, 2002; Shahidi, 1997). Therefore, antioxidant agents are inevitably used for maintaining the quality of this group of bakery products.

Synthetic antioxidants such as BHT, BHA, and TBHQ are used extensively in commercial bakery products to maintain the oxidative quality of oils and fats (Dziezak, 1986). Nowadays, however, there is an increased concern about the consumption of synthetic additives and their effects on consumer health (Razali et al., 1997; Tsuda et al., 1994). Another problem with synthetic antioxidants is that they are volatile at high temperatures and decompose easily, thereby limiting their use in products which receive high temperatures through baking or processing (Martinez‐Tome et al., 2001). The food industry, especially the bakery industry, is interested in using natural antioxidant that is effective, economical, and which pose no health risks, as compared to synthetic antioxidants (Bhanger et al., 2008; Delvarianzadeh et al., 2020). Natural antioxidants are generally thought to be harmless because of their inherent qualities of being secondary metabolites in plants (Bialek et al., 2016). One of the most important sources of natural antioxidants is medicinal plants. They are good candidates for researchers and food industries, especially when the aim is to select natural antioxidants for usage in food products (Chang et al., 2016; Daneshzadeh et al., 2019). This is mainly because of their favorite flavor, healing attributes, antimicrobial potential, and antioxidant properties. There are numerous reports on the use of various medicinal plants and their derivatives, for example, essential oils, aqueous or alcoholic extracts, and powders that are obtained from various plant parts (Abdel‐Samie et al., 2011; Amany et al., 2012; Badei et al., 2002; Chang et al., 2016; Delvarianzadeh et al., 2020; Isobe et al., 2004; Izzreen & Noriham, 2011; Kanatt et al., 2007; Kozłowska et al., 2014; Mildner‐Szkudlarz et al., 2009; Mohammadhosseini et al., 2016). Phenolic and flavonoids constituents are major compounds in medicinal plants that have antibacterial, antitumor, and antioxidant properties, while having the ability to increase the shelf life of food products (Aslanipour et al., 2017; Nychas et al., 2003).

Lemon verbena (*Lippia citriodora* H.B.K.) is a medicinal and aromatic plant with wide usage belongs to Verbenaceae family. Lemon verbena is an annual plant that originates in South America and is nowadays grown commercially in North Africa (Morocco), Southern Europe, and various parts of Iran (Casamassima et al., 2013). The plant contains various phenolic and flavonoid compounds that validate its use as a natural antioxidant (Naser Aldeen et al., 2015). It has traditionally been used as tea seasoning and herbal tea due to its pleasant aroma and taste (Pascual et al., 2001). Therefore, it seems that lemon verbena can be used for enriching cookies and can be acceptable by consumers.

Accordingly, the objectives of this study are a) to study the phytochemical properties of lemon verbena and identify the chemical composition of essential oil (the plant growth in Iran), as well as its alcoholic extract, via GC/MS and HPLC, respectively; b) to determine the antioxidant activity of lemon verbena essential oil via the DPPH method and by drawing a comparison with the synthetic antioxidant TBHQ; and c) to evaluate and compare the use of essential oil and lemon verbena powder in cookies, their physicochemical and sensory properties and oxidative stability of cookies fat for a 6‐month period of shelf life.

## 2 MATERIALS AND METHODS

### 2.1 Materials

The raw materials for making cookie dough were wheat flour, margarine, whole egg, sugar, vanilla, skim milk, salt, and baking powder, purchased from a local supplier. The dried leaves of lemon verbena (*L. citriodora* H.B.K.) were harvested from a horticultural plant research center (HPRC) of Jahrom University (Jahrom, Iran). Other chemicals and solvents were of analytical grade, purchased from Sigma‐Aldrich Company Ltd. (Gillingham, UK).

### 2.2 Methods

#### 2.2.1 Plant preparation

One batch of Lemon verbena (*L. citriodora*) leaves was obtained from the HPRC in the harvest season (November 2019). Immediately after harvest, fresh lemon verbena leaves were cleaned. Healthy leaves were selected to be included in sample preparation. The plant was dried at ambient temperature (28 ± 2°C) for 72 hr.

#### 2.2.2 Essential oil (EO) extraction

Shade‐dried leaves of lemon verbena were subject to water distillation for 3 hr in an all‐glass apparatus, which eventually generated yellow essential oil. The Clevenger method was used for extracting the essential oil from the lemon verbena leaves. The isolated oil was dried over anhydrous sodium sulfate and stored in a laboratory freezer (−18°C). The extraction efficiency was calculated based on the dry weight of leaves (v/w).

#### 2.2.3 Plant extraction and biochemical measurements

Plant materials were extracted according to the maceration method, as described by Wojdyło et al., (2007) with some minor modifications. The antioxidant activity of the extract was determined by a spectrophotometric method based on the reduction of a methanol solution from DPPH (Oke et al., 2009). Assays on flavones and flavonols were carried out according to Popova et al., (2004), with some minor modifications, and the results were reported as mg quercetin/g dry weight of plant sample (Popova et al., 2004). Total flavonoid content was calculated according to the formation of a flavonoid–aluminum complex, while quercetin was selected as a standard (Menichini et al., 2009). Total phenolic contents were analyzed using the Folin–Ciocâlteu colorimetric reagent, and gallic acid was used as a standard (Wojdyło et al., 2007).

#### 2.2.4 Gas Chromatography/Mass Spectrometry (GC/MS) Analysis

Identifying the components of essential oils involved using the gas chromatography (GC) and gas chromatography‐mass spectrometric (GC‐MS) analyses. To identify the constituents of the lemon verbena essential oil, an Agilent Technologies‐7890A gas chromatograph device was used. The type, length, diameter, and thickness of the column were HP‐5‐MS, 30 m, 0.32 mm, and 0.25 μm, respectively. The temperature program of the column was set to change within the range of 60–210°C at a rate of 4°C/min. Nitrogen carrier gas was used at a flow rate of 0.5 ml/min. The gas chromatograph was connected to a mass spectrometer (GC‐MS) (Agilent Technologies‐5975C). The column type was HP‐5MS, being 30 m in length, 0.25 mm in diameter, and 0.25 μm in thickness. The temperature program was 280°C, and helium carrier gas was used at a flow rate of 1 ml/min. The relative percentage of each component of EO was determined based on chromatogram peak area and compared with the total area by using the normalization method of the GC/FID peak areas. Retention indices were determined using retention times of n‐alkanes (C_8_‐C_25_) that were injected after the volatile oil under the same chromatographic conditions. The retention indices for all components were determined by using n‐alkanes as standard. Identifying the spectra involved using a data bank of mass, retention time, Kovats index, and a study of mass spectra per essential component and pattern of spectral refraction, compared with standard spectra and with the use of reputable sources (Adam, 2001).

#### 2.2.5 Identification of polyphenol by HPLC

Extraction, separation, and quantification of phenolic compounds were performed according to method of Mišan et al., (2011) with some modifications. Plant extracts were prepared by macerating 200 mg dried samples with a solution of methanol/acetic acid mixture (85:15) for 24 hr at 4°C and subsequently extracted in an ultrasonic bath at room temperature for 15 min. The resulting suspension was then centrifuged at 10,000 rpm for 20 min at 0°C. To remove compounds such as chlorophylls and lipids, the supernatant was extracted with 1 ml n‐hexane and centrifuged at 10,000 rpm for 10 min. After removing the supernatant, the resulting solution was used for the analysis of both total phenolic contents and their components. The HPLC system employed consisted of a high‐performance liquid chromatography (Agilent 1,200 series) equipped with a UV‐Vis multi‐wavelength detector at 280 and 330 nm. Data were evaluated using a ChemiStation Software (Agilent Technologies) data processing system. The separation of components was achieved by an Agilent, XDB‐C18, 5 μm, 4.6 × 150 mm column, at a flow rate of 1 ml/min. Solvent gradient was performed by varying the proportion of solvent A (methanol) to solvent B (2% acetic acid in water) to separation chlorogenic acid in 330 nm and other compounds in 280 nm. The total running time and postrunning time were 30 and 10 min, respectively. The column temperature was 30°C. The volumes of samples and standards injected were 20 μl which was done automatically using autosampler (Mišan et al., 2011).

#### 2.2.6 Dried leaves powder preparation

Lemon verbena leaves were shade‐dried at room temperature (28 ± 2°C). The dry lemon verbena leaves were ground and powdered by a kitchen blender before being mixed in the cookie dough.

#### 2.2.7 Cookies preparation

The cookie doughs were made in 8 formations, that is, 0.5 (F1), 1 (F2), and 1.5% (F3) dry leaf powder, 200 (F4), 2000 (F5), and 5,000 ppm (F6) lemon verbena essential oils, TBHQ (200 ppm) (F7), and without additive (control) (F8). The cookies were prepared according to a recipe described in the AACC method, with slight modifications (AACC, 2000). Crystalline sugar (250 g) was mixed with shortening (250 g) for 8 min in order to produce creamy batter. The dry ingredients including skimmed milk (10 g), salt (1.5 g), baking powder (6 g), and wheat flour (500 g) which were added to the creamy batter. Then, beaten whole eggs (75 g) were mixed with water (100 g) and then added to make dough. The dough was divided accurately to make each piece 20 g in weight. It was then rounded and sheeted into cookie shapes (5 cm diameter with 3 mm thickness) before baking at 180°C for 20 min in a convection oven. The cookies were packed in polyethylene bags and stored at room temperature (25°C) for 6 months. The cookie samples were examined after 0, 2, 4, and 6 months of storage.

#### 2.2.8 Extraction of fats

The cookies were ground and their fat content was obtained, as described by Hallabo (1977) via cold extraction with n‐hexane. About 100 g of each sample was shaken with 200 ml n‐hexane in a metabolic shaker for 24 hr before being filtered. In a fat‐solvent mixture, the n‐hexane evaporated after filtration at room temperature by a vacuum pump under a laboratory hood. The fat residues were obtained after being dissolved and were relocated to a glass tube for storage at freezing temperature (−18°C) for further analysis.

#### 2.2.9 Oxidative stability

The stability of cookie's fats was examined periodically at intervals of 60 days during the 6 months of storage at ambient temperature. The assessments involved pH, acid value, peroxide value, p‐Anisidine value, and TOTOX value, according to previous methods described by AACC (2000) and A.O.C.S. (1993).

#### 2.2.10 Moisture content

Moisture content was determined by gravimetric heating (130 ± 2°C for 3 hr) using a 5 g ground cookie sample on a preweighed petri plate, according to the 44–15 AACC method (AACC, 2000). After heating, petri plates cooled in a desiccator at room temperature and the loss of weights were reported as moisture content (%).

#### 2.2.11 Baking loss and weight loss

Baking loss (%) was calculated as the difference between the weight of the cookie before and after the baking process. Weight loss was determined by weighing cookies after 2, 4, and 6 months of storage. All measurements were carried out in triplicate.

#### 2.2.12 Sensory evaluation

Cookie samples were evaluated for their sensory characteristics on a five‐point hedonic scale. This involved assessing color, appearance, flavor, taste, texture, mouth feel, and overall acceptability. Ten trained panelists volunteered to test the samples.

#### 2.2.13 Statistical analysis

Experimental and organoleptic data were analyzed for variance (ANOVA) and significant differences were identified according to Tukey test (HSD: High significant differences) (*p* <.05), using the JMP 8.0 software. The analyses were carried out in triplicate, and the results were presented as average values.

## 3 RESULTS AND DISCUSSION

### 3.1 Biochemical analysis

According to the results, the inhibitory power against DPPH radicals in lemon verbena extract was 77.35 ± 0.21%. In this regard, Farahmandfar et al., (2018) reported that increasing the concentration of lemon verbena essential oil from 200 to 3,200 ppm in sunflower oil caused the free radical scavenging activity of DPPH to increase from 10% to 56%. Furthermore, Choupani et al., (2014) reported that the level of DPPH radical scavenging activity by the methanol extract of lemon verbena was 85% and that this amount was higher than the ethanol, acetone, and aqueous extracts.

A variety of phytochemical compounds in medicinal plants exhibit an array of protective and therapeutic properties that function effectively and are necessary for preventing the occurrence of disease. One group of these phytochemical compounds is phenolic compounds. The results showed that the amount of total phenolic compounds in the methanol extract of lemon verbena leaf was 49.2 ± 0.11 mg/g (Table 1). This was higher than the amount reported in a study by Choupani et al., (2014). The researchers reported that the total phenolic compounds in the methanol, ethanol, acetone, and aqueous extracts of lemon verbena were 25.94, 23.75, 18.06, and 16.58 mg/g, respectively. Mohtashami et al., (2013) reported that one of the factors affecting the amount of total phenolic compounds in medicinal plants is the phenological stages and growing conditions of the plant. Accordingly, they reported that the amount of phenolic compounds in the total methanol extract of *Dracocephalum moldavica* differed in the vegetative, floral stages budding, full flowering, and fruiting set, being 189, 145.2, 225.5, 211.5, and 192.8 mg/100 g in field conditions and 15.8, 109.1, 126.3, and 133.1 mg/100 g in greenhouse conditions, respectively. The results of the current research suggest that the amounts of total phenolic compounds in plant biomass are similar to previous results reported by Naser Aldeen et al., (2015), as the levels of total phenolic compounds in lemon verbena differed at various stages of growth and ranged from 22.83 ± 0.76 to 48.21 ± 1.35 mg/g based on gallic acid. Extraction method can also affect the amount of phenolic compounds measured.

**TABLE 1:** Biochemical analysis of methanol extract of lemon verbena leaves (*L. citriodora*)

Sample name	Total phenols content (mg Gallic acid/g)	Total flavonoid content (mg Quercetin/g)	Antioxidant activity (DPPH) (%)	Flavones and flavonols content (mg Quercetin/g)
Methanol extract	49.2 ± 0.11	14.54 ± 0.67	77.35 ± 0.21	2.23 ± 0.08

Total flavonoids, and flavone and flavonols in the methanol extract of lemon verbena were 14.54 ± 0.67 and 2.23 ± 0.08 mg/g quercetin, respectively (Table 1). Naser Aldeen et al., (2015) reported that the total flavonoids in the leaves of lemon verbena varied at different stages of growth and ranged from 2.41 ± 0.07 to 7.56 ± 0.1 mg/g based on quercetin. They discovered that various factors such as genetics, ontogenetic, biotic and abiotic factors, and plant organ type have significant effects on the amounts of secondary metabolites and compounds in plants, including flavonoid compounds. In the current study, the total flavonoid content of lemon verbena appeared to be higher than those reported in similar studies. The difference is probably related to the conditions of growth (weather temperature and humidity), phonological stage, harvest time, soil nutrition, etc.

### 3.2 Gas chromatography–mass spectrometry (GC/MS) analysis

The gas chromatogram analysis showed that there were 41 different compounds in lemon verbena essential oil, which comprised 95.24% of the total essential oil (Table 2). The most important components of lemon verbena were found to be geranial (27.21%), neral (20.01%), spathulenol (7.28%), limonene (6.86%), caryophyllene oxide (6.77%), α‐curcumene (4.64%), and 1,8‐cineole (4.61%). In previous cases of research on the components of lemon verbena essential oil, Argyropoulou et al., (2007) reported that the number of components in the essential oil of this plant differed at two important stages, that is, the vegetative stage and the flowering period, and were 43 and 42, respectively. The most important components of lemon verbena essential oil in the vegetative stage and the flowering stage were geranial (38.7% and 26.8%), neral (24.5% and 21.8%), and limonene (5.8% and 17.7%), respectively. Different amounts of spathulenol (3.1%) and α‐curcumene (2.5%) were also measured in lemon verbena essential oil (Argyropoulou et al., 2007). Also, Nourafcan et al., (2014) indicated that the most important components of lemon verbena essential oil were neral, geranial, limonene, and 1,8‐cineole, when the plants grew in Iran. The difference in the amounts of lemon verbena essential oil components in this study, as compared with previous studies, can probably be attributed to climatic conditions of the region, soil nutrients, and plant growth stage at the time of sampling (Moradi et al., 2014).

**TABLE 2:** Volatile compounds identified in lemon verbena (*L. citriodora*) essential oil

No.	Compounds	RI	Percentage	No.	Compounds	RI	Percentage
1	α‐Pinene	933	0.268	22	Nerol	1,228	0.225
2	Sabinene	973	0.561	23	Neral	1,239	**20.005**
3	1‐Octen−3‐ol	976	0.561	24	Carvone	1,244	0.243
4	6‐methyl−5‐Hepten−2‐one	986	1.914	25	Geraniol	1,254	0.399
5	Myrcene	990	0.096	26	Geranial	1,267	**27.206**
6	*n*‐Decane	1,000	0.130	27	α‐Copaene	1,375	0.407
7	α‐Terpinene	1,017	0.044	28	Geranyl acetate	1,384	1.438
8	p‐Cymene	1,024	0.143	29	α‐Cedrene	1,411	0.343
9	Limonene	1,028	**6.860**	30	(E)‐Caryophyllene	1,419	1.562
10	1,8‐Cineole	1,031	**4.612**	31	α‐Humulene	1,453	0.097
11	(E)‐β‐Ocimene	1,046	0.363	32	Allo‐Aromadendrene	1,460	0.416
12	δ‐Terpinene	1,057	0.139	33	δ‐Muurolene	1,480	0.340
13	cis‐Sabinene hydrate	1,066	0.112	34	ar‐Curcumene	1,483	**4.636**
14	Linalool	1,099	0.156	35	δ‐Cadinene	1515	0.671
15	Perillene	1,101	0.582	36	γ‐Cadinene	1524	0.178
16	Citronellal	1,153	0.584	37	(E)‐Nerolidol	1564	0.883
17	γ‐Terpineol	1,166	0.207	38	Spathulenol	1577	**7.275**
18	Terpinen−4‐ol	1,177	0.598	39	Caryophyllene oxide	1582	**6.767**
19	α‐Terpineol	1,190	1.412	40	epi‐α‐Cadinol	1,640	1.002
20	Methyl chavicol	1,199	0.224	41	α‐Cadinol	1653	1.266
21	trans‐Carveol	1,219	0.314				

RI: The retention Kovats index were determined on HP‐5 capillary column.

Components less than 0.05% was not reported.

### 3.3 Qualitative–quantitative high‐performance liquid chromatographic analysis

The different components of lemon verbena extract were fractionated and identified via the HPLC technique. In total, 17 different standards were injected, and phenolic compounds were detected at 280 nm. Trans‐ferulic acid, hesperidin, and ρ‐coumaric acid were identified by making a comparison between the inhibition time, which was detected in the chromatogram, and the illustration of standard curves. According to HPLC analysis, the most important components of lemon verbena extract were trans‐ferulic acid (6.71 mg/g), followed by hesperidin (1.87 mg/g), whereas the least significant ingredient in lemon verbena extract appeared to be ρ‐coumaric acid (0.04 mg/g). More details of the components in lemon verbena extract are presented in Table 3. Bilia et al., (2008) reported that the most important compounds identified in the ethanol extract of lemon verbena by HPLC/DAD/ESI/MS were verbenalin, verbascoside, isoverbascoside, and eukovoside. Their results and ours differed probably because of variations in the type of standards used.

**TABLE 3:** Polyphenol constituents identified by HPLC method in *L. citriodora* leaves cultivated in Iran

Compound name	Content (mg/g dry weight)	Retention time (min)	Wavelength (nm)
Gallic acid	nd	3.30	280
Catechin	nd	8.30	280
Chlorogenic acid	nd	10.50	320
Rutin	nd	12.40	280
Vanillin	nd	13.50	280
ρ‐Coumaric acid	**0.041**	15.86	280
Trans‐Ferulic acid	**6.710**	16.93	280
Cumarin	nd	17.40	280
Hesperidin	**1.866**	18.36	280
Ellagic acid	nd	19.02	280
Rosmarinic acid	nd	19.20	280
Eugenol	nd	23.70	280
α‐Tocopherol	nd	24.96	280

Abbreviations: Nd, Nondetected; Nm, Nanometer.

### 3.4 Thermal stability of cookie's fat

**FIGURE 1:**
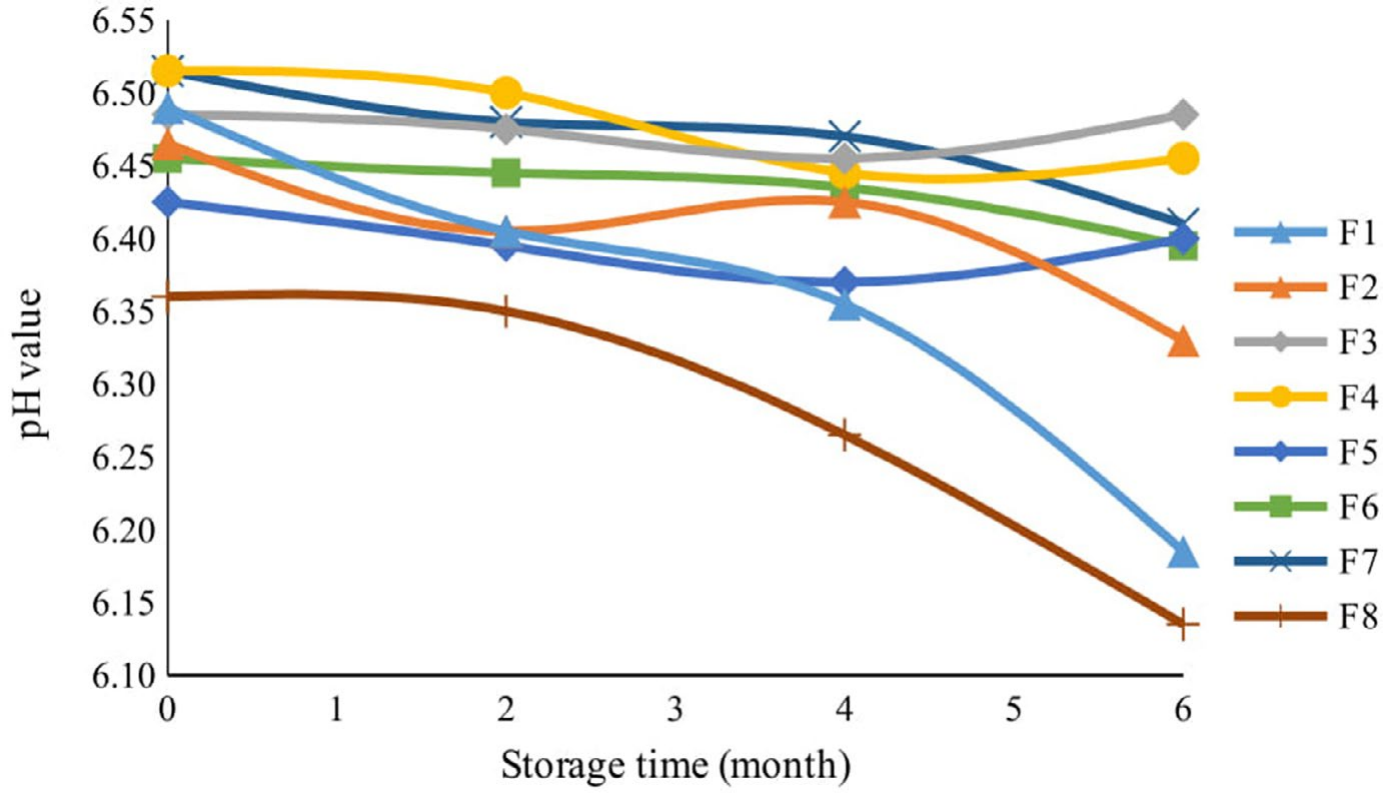
The effect of storage time on pH value of cookies with different formulations

Fat content was extracted from cookies and evaluated for pH, acid value, peroxide value, p‐Anisidine value, and TOTOX value to determine the functionality of various concentrations of essential oil and lemon verbena powder in comparison with TBHQ. The results according to variance analysis showed significant effect of treatment on measured factors (Table 4). Changes in pH were directly related to the amount and the release rate of free fatty acids, as well as the buffering properties of components in the cookie formulation. According to Figure 1, there was no significant difference between the pH of different formulations, apart from the pH of the control sample after baking (*p* <.01). The pH of the control sample was the lowest of all after baking, which indicates fatty acid degradation and the breakdown of triglycerides which generated free fatty acids during the baking process. Through storage, there was a gradual decrease in the pH of samples containing essential oil and lemon verbena powder, similar to samples containing the synthetic antioxidant TBHQ. After the control sample, the sharpest decline in pH value occurred in F1 and F2 formulations during the 6 months of storage. In comparison, slighter levels of decline in pH occurred in formulations F3, F4, F5, and F6. There was a strong correlation between the changes in pH and the acid value of fat component in different cookie formulations (Table 5). Determining the pH index can be a quick way to confirm the progress of spoilage in fats. As mentioned, however, the buffering properties of ingredients in the food formulation make this method unreliable if it were to be used alone. Thus, a successful confirmation of the pH results required assessments of acid value, peroxide value, p‐Anisidine value, and TOTOX value, all of which were performed herein.

**TABLE 4:** Analysis of variance of physicochemical properties of cookie's fat

SOV	DF	Weight loss	MC	pH	Acidity	PV	p‐Anisidine	TOTOX
Time	3	80.37**	77.75**	0.057**	0.032**	22.73**	108.14**	194.52**
Treatment	7	2.54**	26.61**	0.057**	0.018**	12.91**	6.93**	85.86**
Time*Treatment	21	0.62**	0.62**	0.007**	0.003**	1.74**	0.07**	6.57**

Abbreviations: DF, Degree of freedom; ns, not significant; PV peroxide value; SOV, Source of variance.

* *p* <.05

** *p* <.01.

**TABLE 5:** The correlation coefficient between most important properties of cookie's fat

	pH	Acid value	Peroxide value	P‐Anisidine value
Acid value	−0.892**			
Peroxide value	−0.203ns	0.345ns		
P‐Anisidine value	−0.719*	0.683*	0.318ns	
TOTOX value	−0.439ns	0.538ns	0.941**	0.619*

Note

Pearson's correlation test was used for correlation relationship calculation.

Abbreviation: ns: Not significant correlation

* Significant correlation in levels of 5%;

** Significant correlation in levels of 1%.

**FIGURE 2:**
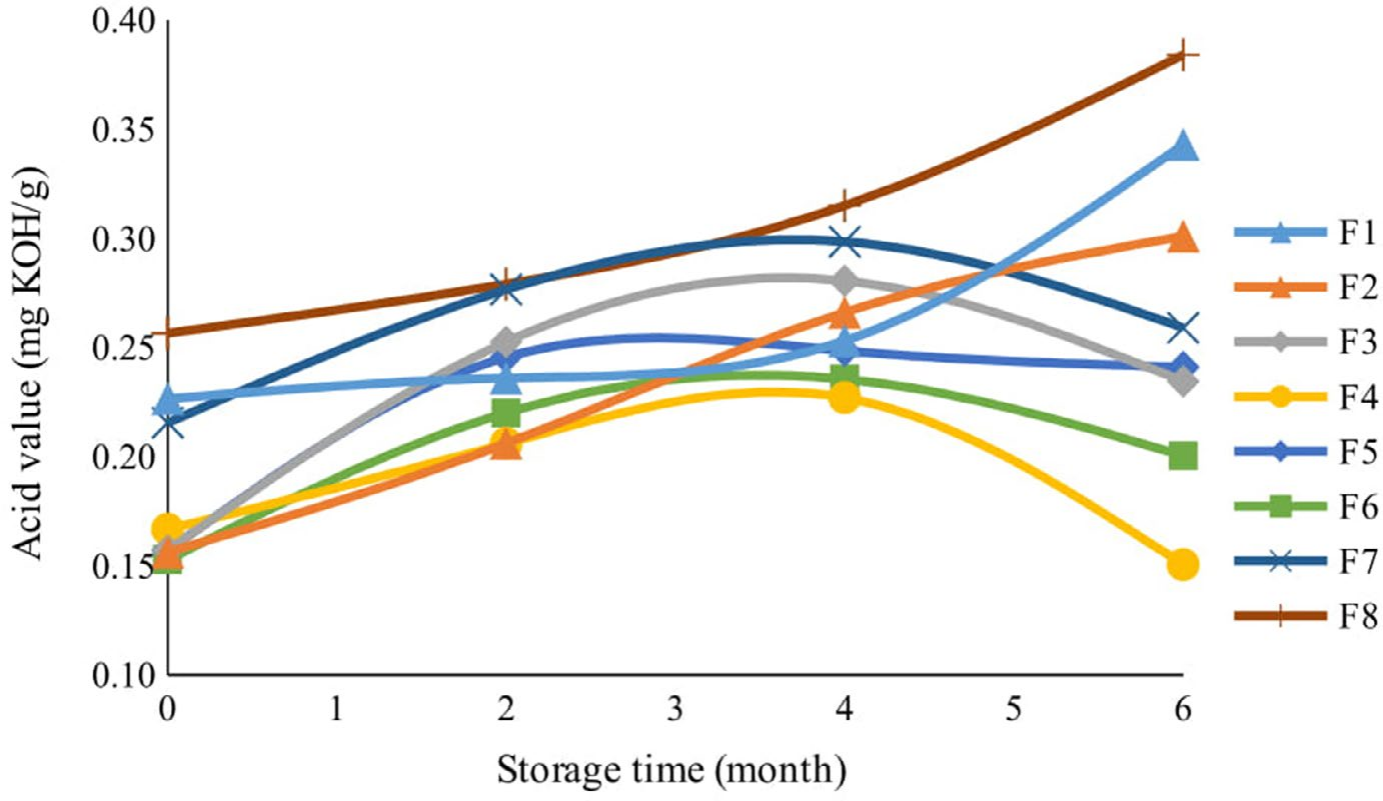
The effect of storage time on acid value of cookies with different formulations

According to Figure 2, there was a significant difference between the acid value of fats extracted from different cookie formulations produced with different amounts of essential oil, lemon verbena powder, and TBHQ after baking (*p* <.01). In fact, after baking the cookies, the highest acid value was measured in the control sample, whereas low acid values were measured in samples F2, F3, F4, F5, and F6, thereby showing how lemon verbena essential oil and powder can hamper the thermal decomposition of fat content in cookies. Up to the fourth month of storage, the acid value gradually increased in all cookie formulations. From the fourth to the sixth month, this index decreased in formulations F3, F4, F5, F6, and F7, whereas it increased significantly in formulations F1, F2, and F8. The decline in acid value in the above formulations is probably related to the reduction of cookie moisture during storage and, thus, a decrease in the hydrolysis rate of triglycerides. Free fatty acids are formed when triglycerides react with water and cause hydrolysis (Frega et al., 1999). On the other hand, high concentration of essential oil and verbena powder are naturally associated with high antioxidant activity that hinders the hydrolysis of triglycerides and slows down the production of free fatty acids. The increase in acid value in formulations F1 and F2, which contained lower amounts of lemon verbena powder, can be explained by the gradual disappearance of bioactive compounds in the cookie during storage. This is accompanied by a decrease in inhibitory effects against oxidation and rancidity when less amounts of phenolic compounds are present. The protective ability of the bioactive ingredients in lemon verbena to hamper the hydrolysis of triglycerides and the production of free fatty acids in cookies during storage was greater than the ability of essential oil and powder obtained from cardamom and cinnamon in cookies (Badei et al., 2002), drumstick (*Moringa oleifera*) (Reddy et al., 2005), amla leaves (*Emblica officianalis*), raisins (*Vitis vinifera*) in biscuits, and rice bran extract in cookies (Bhanger et al., 2008).

**FIGURE 3:**
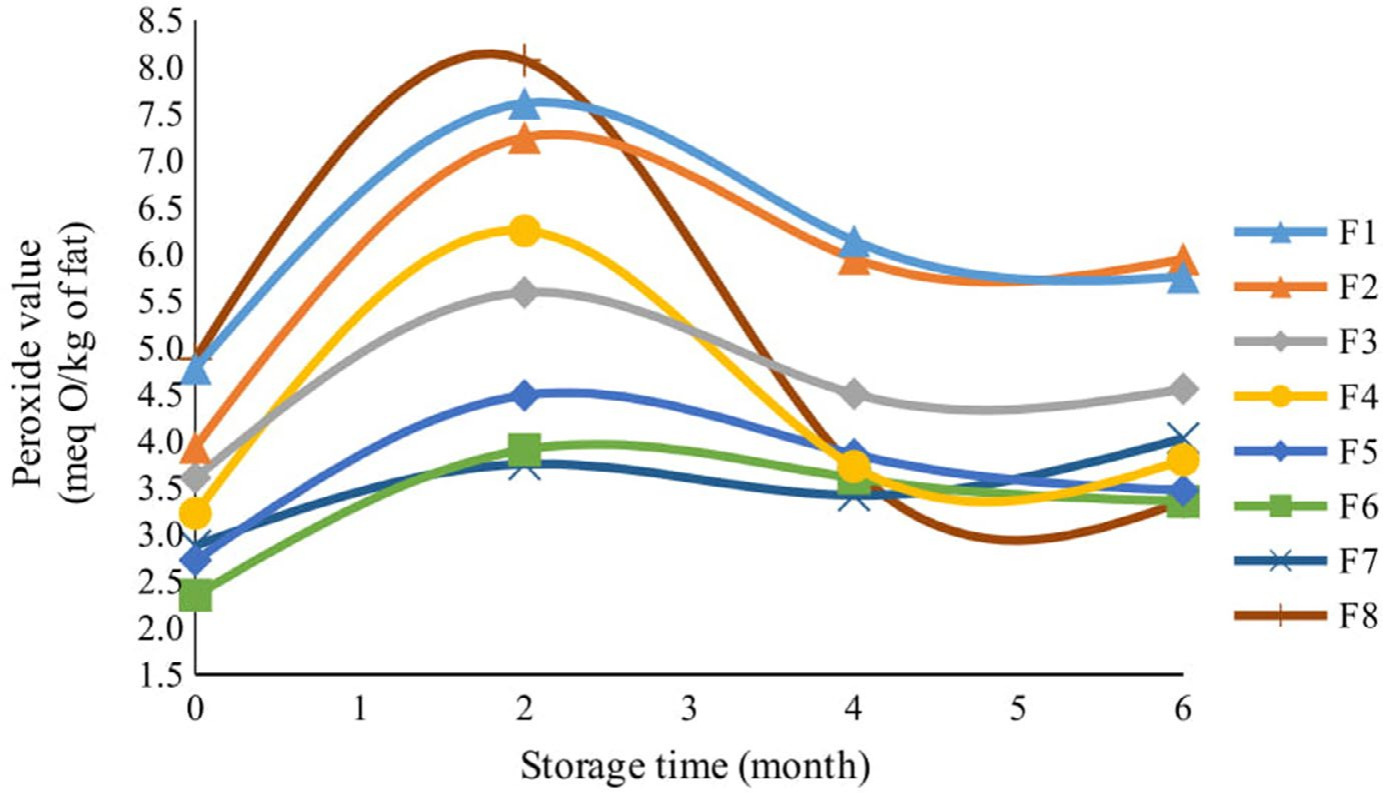
The effect of storage time on peroxide value of cookies with different formulations

There were variations in the changes that occurred to the peroxide values of different cookie formulations (Figure 3). The results showed that the peroxide value of the control sample was highest among other samples after baking (*p* <.01). Formulations containing high concentrations of lemon verbena powder (F3), lemon verbena essential oil (F4, F5, and F6), and TBHQ had the most effective protection and slowed down the increase of peroxide value after baking. The control sample, however, had the highest peroxide value at all times of storage. In the case of samples containing lemon verbena powder in small amounts (F1 and F2), a trend of increase was observed in the peroxide value during the first two months of storage, but thereafter the peroxide value decreased in these samples (*p* <.01). In all samples, after baking, the amount of peroxide value increased during the first two months and then decreased (Figure 3). Compared to the control sample, the effective compounds of lemon verbena exhibited antioxidant effects in all samples containing the essential oil and powder. The oxidation of fats was favorably delayed, and this was predictable according to the analysis of the essential oil and verbena extract, as revealed by the GC/MS and HPLC (Tables 2&3). Hydroperoxides are the primary oxidation products of fats. They are odorless and colorless but are highly volatile and can be broken down into more complex products including ketones, aliphatic aldehydes, alcohols, and hydrocarbons, collectively known as secondary products of oxidation (Kolakowska, 2003). Therefore, the safety of food products that contain fat is calculated based on the progress of oxidation processes. Tracking and measuring primary and secondary products of fat oxidation can help control the quality of bakery products, but because the destruction and change of primary and secondary products of fat oxidation are done continuously over time, measuring one of these parameters alone cannot yield accurate results of the fats oxidation intensity. Thus, primary oxidation products (the peroxide value being an indicator) and secondary oxidation products (the p‐Anisidine being an indicator) are measured simultaneously. This can serve as an optimum way to control the oxidation of fat products (Bialek et al., 2016; Pegg, 2001).

When the peroxide value is between 10 and 20 meq./kg of fat, the food has technically become rancid but is still acceptable in terms of flavor and taste. However, if this number exceeds 20 meq./kg, the food becomes no longer acceptable by the consumer because the strong rancidity affects the food flavor as well (Pearson, 1970). On the other hand, the maximum peroxide value being allowed in a cookie is 2 meq./kg, but because in this study, the amount of fat in the cookies was about 20%, the allowable peroxide value in the fat content of cookies becomes 10 meq./kg. During the 6 months of cookie storage at room temperature, the peroxide value of all samples was below 10 meq./kg, which indicates the oxidative stability of the essential oil and the lemon verbena powder. Badei et al., (2002) reported that the peroxide value reached no more than 10 meq./kg in cookies containing the essential oils and powders of cardamom and cinnamon during a 6‐month shelf life, similar to the results of our study. Phenolic extracts in plant tissues can have inhibitory effects on the peroxide value in bakery products. In this regard, adding 1,000 mg of phenolic chokeberry extract reduced the peroxide value of cookies through 18 weeks of storage (Bialek et al., 2016). In the same report, it was claimed that the highest peroxide value was observed in the ninth week of storage, after which time the peroxide value decreased significantly in the cookies.

**FIGURE 4:**
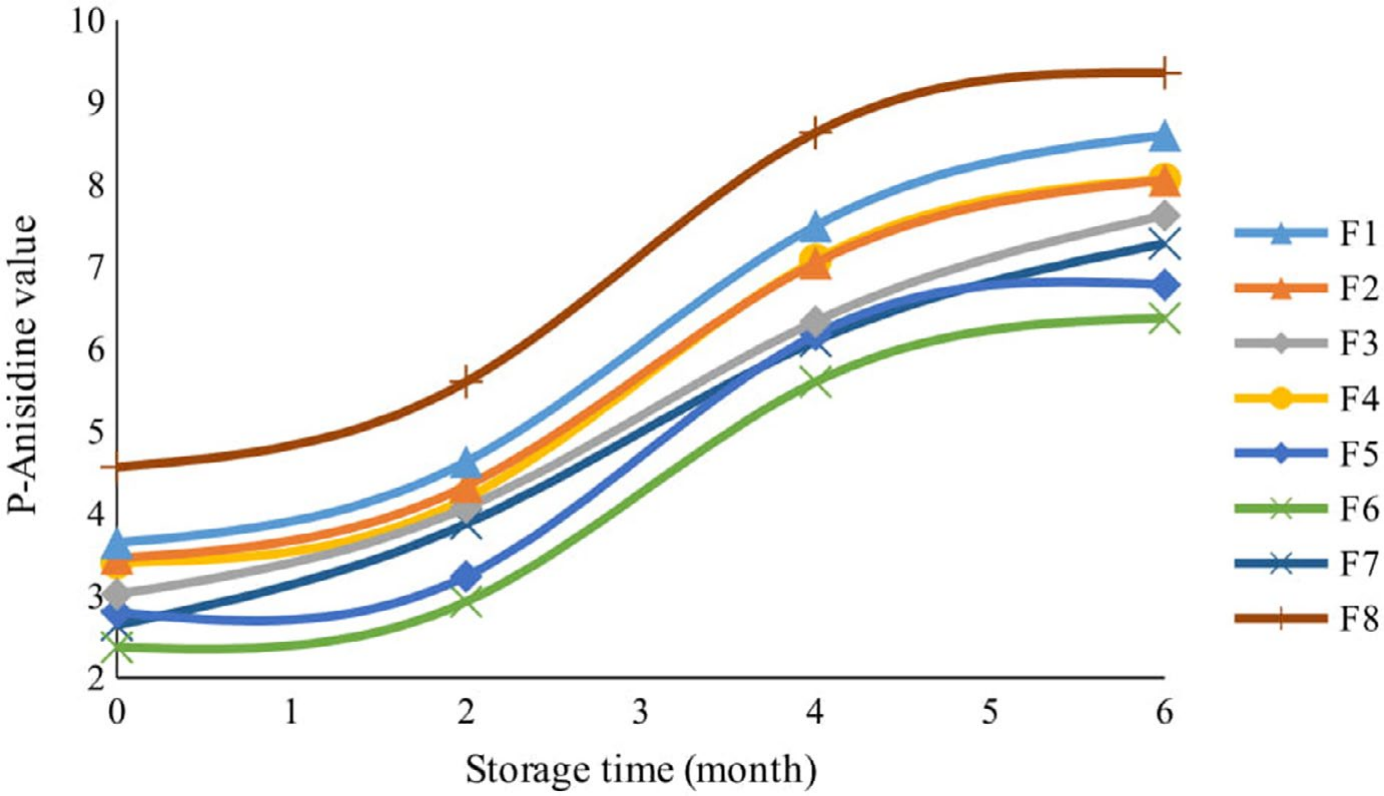
The effect of storage time on p‐Anisidine value of cookies with different formulations

The peroxide value is generally used for determining the amount of oxidative degradation in oils, fats, and fatty foods. However, the rapid conversion of hydroperoxides to secondary oxidation products makes the peroxide value an incomplete criterion for determining the oxidative degradation of oils. To yield more accurate results, it is recommended that other quality tests be carried out on oils and fats (Bhanger et al., 2008). One of the most valid tests that can determine the oxidative stability of oils and fats is the p‐Anisidine test. In all cookie samples, the p‐Anisidine value increased significantly over time (*p* <.01). While considering that the p‐Anisidine value represents the amount of secondary oxidation compounds, the decrease in peroxide value from the second month of storage onwards occurred parallel to an increase in the p‐ Anisidine value (Figure 4). During the 6 months of storage, p‐Anisidine levels increased from 3.02 to 8.60 in cookies that contained lemon verbena powder. In cookies containing lemon verbena essential oil, it increased from 2.37 to 8.06, whereas it increased from 2.64 to 7.28 in cookies containing TBHQ. In the control sample, however, the p‐Anisidine value increased from 4.56 to 9.35. The highest p‐Anisidine value was observed in the control sample after 6 months of storage, whereas the lowest was observed after baking cookies that contained 5,000 ppm lemon verbena essential oil. The analysis of variance showed that ‘time’ and ‘type of additive’, as independent variables, had significant effects on the p‐Anisidine value (*p* <.01), whereas the interaction between these two factors had no significant effect on the p‐Anisidine value. Bialek et al., (2016) reported that the p‐Anisidine value increased from 5.30 to 18.60 and from 4.70 to 22.92 in cookies containing butter and in those containing margarine after 18 months of storage, respectively. Chang, Abbaspour, et al., (2016) reported that the effect of adding 200 ppm of *Foeniculum vulgare* essential oil on reducing the p‐Anisidine value of olive oil was greater than BHT and BHA after 15 days of storage.

**FIGURE 5:**
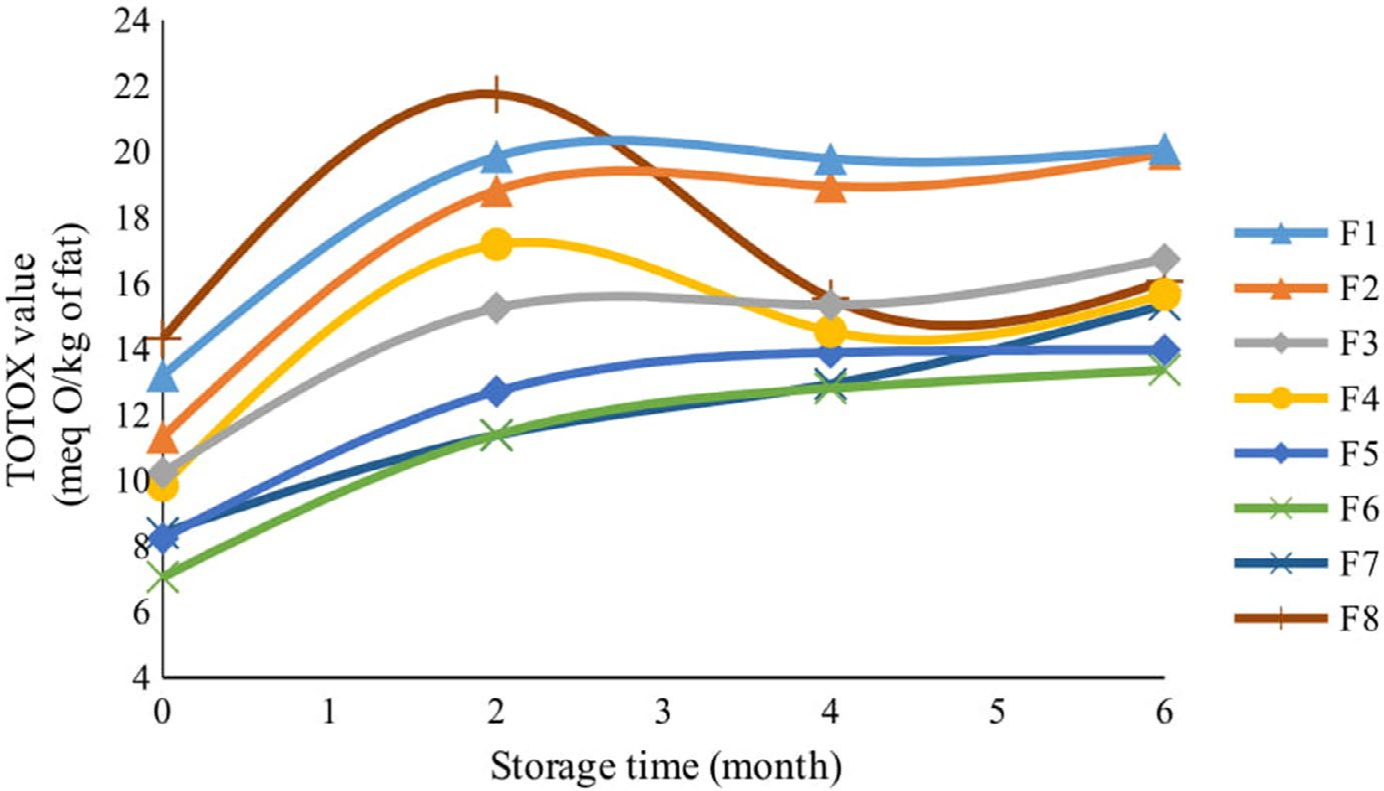
The effect of storage time on TOTOX value of cookies with different formulations

The peroxide value and the p‐Anisidine value represent the intensity of oxidation in the early and final stages of fatty acid oxidation, respectively. The TOTOX value indicates the amount of hydroperoxides and the compounds resulting from the degradation of hydroperoxides. Thus, it is more comprehensive for monitoring the oxidation of fats during storage (Shahidi & Zhong, 2005). In fact, the TOTOX value indicates the amount of carbonyl compounds that are generated in fats, along with other products that form through oxidation during the storage of oils and fats (Mildner‐Szkudlarz et al., 2009). In the current research, changes in the TOTOX value confirmed the changes in the peroxide value and in the p‐Anisidine value altogether (Figure 5). Accordingly, the highest increase in the TOTOX value occurred in the first two months of storage and then increased slightly. The highest amount of TOTOX was related to the control sample, and the lowest was related to cookies containing different amounts of lemon verbena essential oil. Among the samples containing lemon verbena powder, those containing 1.5% powder had the lowest amount of TOTOX through time. The analysis of variance showed statistical significance in the effects of time and type of additive, when considered independently, and also when considered as two interacting factors. Their effects were significant on the changes of the TOTOX value (*p* <.01).

Lemon verbena essential oil has strong antioxidant properties because of its high levels of geranial, neral, spathulenol, and limonene. A collection of these ultimately made the TOTOX value in the cookies lower than the allowable level during the 6 months of storage. Bialek et al., (2016) reported that the level of TOTOX value in cookies containing chokeberry extract increased significantly during 18 months of storage, while changes in the TOTOX value decreased as higher amounts of chokeberry extract were used in the cookies. Mildner‐Szkudlarz et al., (2009) examined the effects of green tea extract on thermal stability and fat oxidation in biscuits. In comparison with the synthetic antioxidant BHA, it was observed that green tea extract has a more significant, positive effect on delaying fat oxidation in biscuits. Thus, the TOTOX values in biscuits containing 1% green tea extract and in those containing 0.02% BHA were 11.40 and 14.01, respectively, during 20 days of storage at 60°C.

### 3.5 Physicochemical properties of cookies during storage

#### 3.5.1 Moisture content

**FIGURE 6:**
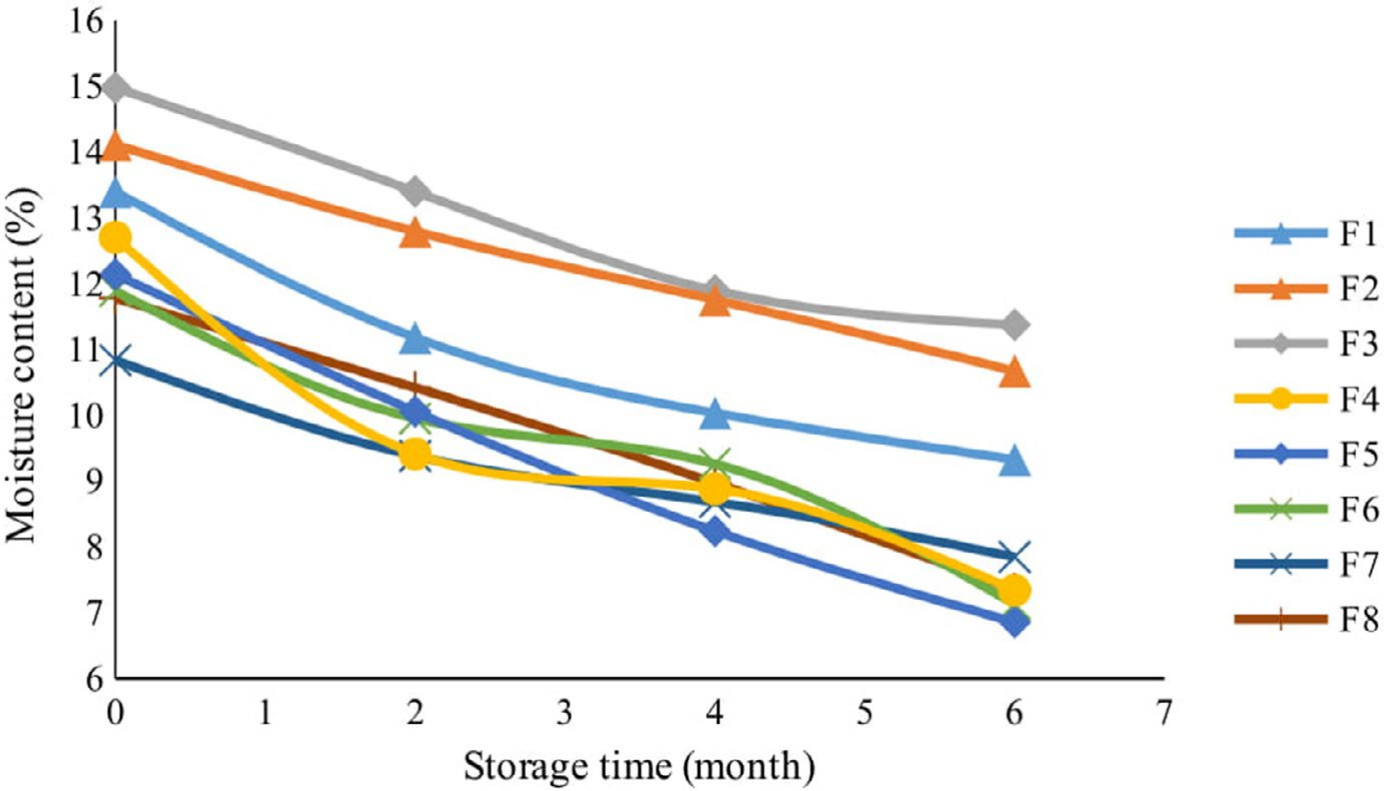
The effect of storage time on moisture content of cookies with different formulations

Variations in cookie moisture contents occurred in response to the application of lemon verbena essential oil and powder through 6 months of storage (Figure 6). According to the results, the moisture content in cookies containing different amounts of lemon verbena powder was significantly higher than the moisture content of other cookies (*p* <.01). Plants usually embody high amounts of soluble and insoluble fiber. Soluble fibers are characterized by high levels of water absorption capacity, and they can absorb most of the moisture inside bakery products to reduce evaporation during the baking process. Therefore, cookies containing high amounts of lemon verbena powder (F5 and F6) had more moisture content than other cookies after baking. In all samples, the moisture content decreased over time. However, this decrease was significantly lower in samples that contained lemon verbena powder. A major problem in bakery products is the movement of moisture from the core of the product to its surface or crust, ultimately leading to moisture loss, which invigorates the staling process. The presence of humectants in bakery products can increase moisture retention and increase the shelf life of products (Pourfarzad et al., 2011). Due to their strong ability to absorb water, plant fibers can act as moisture absorbers and maintain moisture in the product during storage. Therefore, cookies containing higher proportions of lemon verbena powder had more moisture and edibility after 6 months. The analysis of variance showed that the essential oil did not have a significant effect on the moisture content of cookies during storage and, in this regard, there was no significant difference among the different concentrations of essential oil, synthetic antioxidants, and the control group. Sudha et al., (2007) reported that adding 25% apple pomace to cake formulations ultimately improved the moisture content and soluble fiber in cakes, increasing them by 1 and 5.64%, respectively (Sudha et al., 2007). On the other hand, Nanditha et al., (2009) reported that the use of turmeric powder did not have significant effects on the final moisture content of biscuits (Nanditha et al., 2009).

#### 3.5.2 Baking loss and weight loss

The effects of variables were different on baking loss and weight loss during storage. Baking loss denotes the amount of mass released in the form of gas under the influence of heat in the baking process. Higher amounts of baking loss indicate that more moisture is removed from the product through baking, and this leads to a decrease in the shelf life of the baked products (Choi et al., 2007). According to this, the lowest amount of baking loss was observed in samples containing lemon verbena powder (data not shown). Lemon verbena powder, like other plant tissues, contains large amounts of fiber and its soluble fibers are able to absorb large amounts of water. Much of the free water in the cookie formulation is absorbed by the lemon verbena powder and retained in the product while baking. According to results, there is a strong, significant correlation between the moisture content of the cookies after baking and the amount of baking loss. Kim et al., (2012) reported that adding cheonnyuncho powder to sponge cake can increase the initial water holding capacity of dough and reduce the baking loss of cakes.

**FIGURE 7:**
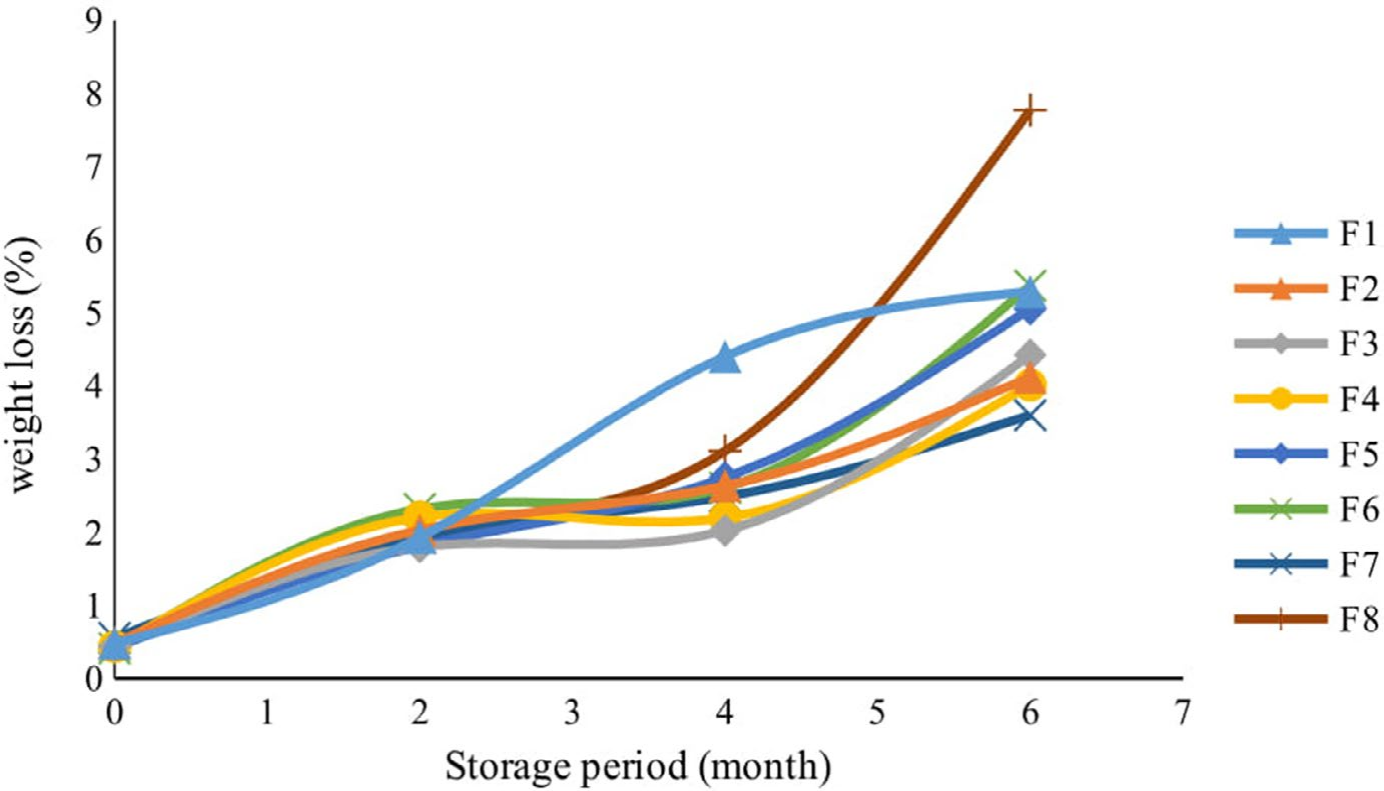
The effect of storage time on weight loss of cookies with different formulations

Different levels of weight loss were measured in the different cookies through storage (Figure 7). Cookies with lemon verbena powder had less weight loss than other samples. The highest rate of weight loss during storage was related to the control sample. There was no significant difference between the weight loss of samples containing lemon verbena essential oil and those containing the TBHQ, as compared to the control sample. Weight loss through storage occurs in response to various factors such as packaging permeability to moisture, vapor pressure of the product, the equilibrium relative humidity of the surrounding atmosphere of product, package volume, storage temperature, etc. (Cauvain & Young, 2009). A lower rate of moisture loss from the product is associated later on with a lower weight loss through storage. An enhanced level of weight loss during storage reduces the shelf life and eating quality of bakery products (Cauvain & Young, 2009). According to Figure 7, the lowest amounts of weight loss through storage were observed in cookies that had the formulations F3, F2, F1, F4, and F7. Plant fibers and their ability to retain high amounts of moisture limited the rate of weight loss in cookies containing lemon verbena powder. The results showed a strong, significant correlation between the rates of weight loss during storage and the rates of change in the moisture content of different cookies.

#### 3.5.3 Sensory evaluation

The cookies scored differently and significantly in terms of sensory evaluation (Table 6). Cookies that contained different amounts of lemon verbena essential oil and powder were studied comparatively along with the control sample and with samples containing TBHQ on the first day of baking and after 6 months of storage. The analysis of variance showed that adding lemon verbena essential oil and powder to the cookies had significant effects on sensory properties. In terms of color, the most acceptable cookies were the F2 and F3 formulations. Adding the green verbena powder to cookies increased the consumer acceptance in terms of color. Nonetheless, there was no significant difference between the scores attributed to the apparent properties of the different cookies, which means that the addition of essential oil or lemon verbena powder did not have other significant effects on the appearance of cookies. The most aromatic cookies, as determined by consumers, were those containing lemon verbena essential oil. Lemon verbena essential oil has a suitable aroma because of its volatile nature and, also, due to the presence of compounds such as limonene, geranial, and neral. By increasing the concentration of essential oil from 2000 to 5,000 ppm, it was observed that the consumers gave significantly higher scores to the flavor feature (*p* <.05). The lowest aroma score was attributed to the control sample and to the cookies containing TBHQ. Samples containing lemon verbena powder also received intermediate scores. As the concentration of lemon verbena powder increased, so did the aroma score but not significant. The sensory evaluation showed that the variables had no significant effect on the textural features of cookies. The scores on texture for all cookie formulations were above 4.5. Adding essential oil to cookies increased their score on taste. The highest taste score was given to 2000 ppm lemon verbena essential oil. As the concentration of lemon verbena essential oil increased from 2000 to 5,000 ppm, the taste score decreased but not significantly. As expected, the lowest taste scores were given to the control group and to the samples with TBHQ.

**TABLE 6:** Mean scores for the sensory properties of cookies enriched by *L. citriodora* essential oil and powders

	Beginning of experiment						
Treatment	Color	Appearance	Flavor	Texture	Taste	Mouthfeel	Overall acceptability
F1	4.7a	4.7a	4.5ab	4.8a	3.6a	4.4a	4.7a
F2	4.9a	4.5a	4.5ab	4.7a	3.7a	4.2a	4.6a
F3	4.8a	4.8a	4.6ab	4.7a	3.7a	4.1a	4.6a
F4	4.5a	4.5a	4.6ab	4.6a	3.5a	4.7a	4.5a
F5	4.6a	4.4a	4.8a	4.7a	3.9a	4.8a	4.9a
F6	4.7a	4.6a	4.9a	4.7a	3.8a	4.9a	4.5a
F7	3.6a	4.6a	3.4c	4.7a	3.5a	4.2a	4.6a
F8	4.8a	4.8a	3.9bc	4.6a	3.4a	4.2a	4.2a
	After 6 month storage						
F1	3.8ab	3.9a	3.5a	2.5a	3.4a	3.3a	3.1a
F2	3.7b	3.8a	3.4a	2.4a	3.3a	3.2a	3.4a
F3	4.0ab	4.3a	3.3a	2.4a	3.4a	2.9a	2.8a
F4	4.2ab	4.1a	3.4a	2.3a	3.5a	3.2a	3.1a
F5	4.3ab	4.2a	3.3a	2.3a	3.4a	3.3a	3.3a
F6	4.7a	4a	3.2a	2.2a	3.3a	3.4a	3.2a
F7	3.5b	3.7a	3.1a	2.5a	3.3a	3.3a	2.9a
F8	4.3ab	4.1a	3.5a	2.4a	3.4a	3.5a	3.0a

Note:

Values with the same lower case letters within each column are not significantly (*p* >.05) different.

Adding lemon verbena powder to the cookies caused a slight increase in scores on mouthfeel, as compared to the control group, but this increase was not significant. The highest score on mouthfeel was given to cookies containing lemon verbena essential oil. By increasing the concentration of lemon verbena essential oil, the score on mouthfeel increased. By increasing the concentration of lemon verbena powder, however, this score decreased. The presence of rough lemon verbena particles which had not been ground well caused a coarse mouthfeel. Secondly, the absorption of free water by plant fibers in the powder made consumers experience a drier mouthfeel while chewing, and so the mouthfeel score decreased in response to higher concentrations of lemon verbena powder in the cookies. In terms of overall acceptability after the first day of baking, the most acceptable formulation was F5 and the worst was F8 (i.e., the control sample).

The results showed that longer durations of storage led to significant effects on all sensory parameters. The sensory score for cookies that were stored for a total of 6 months at room temperature decreased significantly. The most substantial decrease in this score was attributed to the textural properties of the cookies. Parallel to the increase in weight loss and the decrease in moisture content, the cookies became stale and, thus, the texture grew significantly stiff. In evaluating all sensory properties, the storage time had the least effect on the appearance and color of the cookies. As the storage time increased, the flavor and aroma scores of cookies containing essential oil and lemon verbena powder were similar and did not differ significantly. This means that, through time, the flavor and aroma scores decreased faster in cookies containing essential oil than in those containing lemon verbena powder. The volatile nature of essential oils is associated with a high rate of deterioration in their compounds through time, especially when the essential oils are used freely in food formulations. On the other hand, adding aromatic herbal powders, such as verbena powder, is likely to have more stable, enduring effects and turns out to be more successful in maintaining flavor over time. According to the sensory scores, the score on overall acceptance for F3 and F7 cookies was below “3” after 6 months of storage at ambient temperature (Table 6). This indicates that the consumer cannot accept these formulations after the said storage time. The decrease in the overall acceptance score in the F3 formulation was because of a decrease in mouthfeel score. Nonetheless, it can be corrected by thoroughly grinding the verbena powder so as not to leave large, coarse particles in the cookies. Another remedy can be to increase the initial moisture content of the formulation. Similar results were reported by Delvarianzadeh et al., (2020). They reported that all the sensory properties of voluminous bread decreased with increasing *purslane* powder concentration. Amany et al., (2012) reported that the use of medicinal plant essential oils can have positive effects on the sensory properties of bakery products (Amany et al., 2012).

## 4 CONCLUSION

Adding the essential oil, extract, or powder of lemon verbena, at different concentrations, to bakery products containing high amounts of fat can substantially reduce the rate of fat oxidation, as this was observed in several cookie formulations in this study. On the other hand, the presence of aromatic substances in lemon verbena essential oil caused an increase in the sensory properties of the cookies. Our results showed that enriching the cookies with different concentrations of lemon verbena essential oil and powder can significantly increase the oxidative stability of the cookies. The GC/MS, HPLC, and phytochemical tests showed that the main active compounds in the essential oil and extract of lemon verbena are compounds that can inhibit the activity of free radicals and can control the chain of reactions in fatty acid oxidation. The functions and potency of these natural compounds are in fact comparable to those of synthetic antioxidants such as TBHQ, as used in this study. Through storage at room temperature, it was observed that the acid value, peroxide value, p‐Anisidine value, and TOTOX value increased at much slower rates in cookies containing lemon verbena essential oil, as compared to the control sample. At a concentration of 5,000 ppm, lemon verbena essential oil had stronger inhibitory effects on oxidation than what TBHQ did. According to sensory evaluation, cookies containing lemon verbena powder received relatively low scores on acceptance, while cookies containing lemon verbena essential oil received higher scores due to their good flavor and aroma. Compared to synthetic antioxidants, medicinal plants such as lemon verbena can be added to a variety of foods. Their role as healthy additives, along with their functional properties, can deliver strong remedial effects, nutritional properties, and desirable sensory properties. The clinical study of the effect of consuming products containing lemon verbena essential oil on the health of consumers would be worthwhile.

## CONFLICT OF INTEREST

The authors confirm that there is no known conflict of interest associated with this publication. This article does not contain any studies with human participants or animals performed by any of the authors.

## Data Availability

The data that support the findings of this study are available from the corresponding author upon reasonable request.
